# Molecular characterization of phytoplasmas of ‘Clover proliferation’ group associated with three ornamental plant species in India

**DOI:** 10.1007/s13205-016-0558-8

**Published:** 2016-11-11

**Authors:** Ekta Khasa, Gopala, Aido Taloh, T. Prabha, Madhupriya, G. P. Rao

**Affiliations:** 1 Division of Plant Pathology, Indian Agricultural Research Institute, Pusa Campus, New Delhi, 110012 India; 2 Directorate of Floricultural Research College of Agriculture, MPKV, Shivajinagar, Pune, 411005 India

**Keywords:** *Hibiscus rosa*-*sinensis* L., *Saponaria officinalis* L., *Allamanda cathartica* L., Phytoplasma, 16Sr VI-D subgroup, Identification, India

## Abstract

Suspected phytoplasma symptoms of little leaf, yellowing, chlorosis, phyllody, witches’ broom, and stunting were observed on ten different ornamental plant species at New Delhi, Andhra Pradesh, Haryana, Bengaluru, and Pune, India, during March to July 2016. To investigate the possibility of phytoplasma etiology, PCR assays were performed using universal primer pairs (P1/P7 followed by 3Far/3Rev) specific to the phytoplasma *16Sr RNA* gene. First round PCR amplification with primer pair P1/P7 did not yield expected 1.8 kb product of 16S rRNA region from any of the 17 symptomatic samples. However, 1.3 Kb amplicons were observed in nested PCR assays with 3Far/3Rev primer pair in symptomatic leaf samples of *Hibiscus rosa*-*sinensis* L. (Pune isolate), *Saponaria officinalis* L. (Pune isolate), and *Allamanda cathartica* L. (Delhi isolate). No amplifications were observed in any of the other tested symptomatic and non-symptomatic plant samples either in first round or second round of nested PCR assays with phytoplasma specific primer pairs. Pairwise sequence comparison of 16S rDNA sequences of the five positive phytoplasma strains of *A. catharica*, *H. rosa-sinensis,* and *S. officinalis* in the present study revealed 99–100% sequence identities with strains of ‘clover proliferation’ (16SrVI) group. Phylogenetic and virtual RFLP analysis of 16S rDNA sequences of the five identified phytoplasma strains belonging to three ornamental species further confirmed their clustering and grouping with member strains of ‘clover proliferation’ subgroup D. This is the first record of the phytoplasma association of ‘clover proliferation’ subgroup D with *H. rosa*-*sinensis*, *S. officinalis,* and *A. cathartica* in the world.

## Introduction

Phytoplasmas, formerly known as mycoplasma-like organisms (MLOs), are cell wall less Mollicutes that colonize plant phloem sieve tube elements and insects gut wall. They are known to cause devastating losses in crops and natural ecosystems worldwide. They are transmitted from one plant to another by phloem-feeding insects, primarily leafhoppers, plant hoppers, and psyllids (Bertaccini et al. 2014). Phytoplasmas cause diseases in several commercial ornamental plants causing serious economic losses all over the world. Phytoplasma diseases are the major constraint in commercial ornamental plant production by lowering the quantum and quality gaining international importance. Phytoplasma causes different symptoms of general yellowing and stunting of plants, proliferation of shoots, phyllody, virescence, reduced size of flowers in many ornamental plants which affects their economic value (Chaturvedi et al. 2010; Bertaccini 2015). The ‘*Ca. P. asteris*’ 16SrI group is the major group infecting ornamental species worldwide. So far, more than 60 ornamental plant species have been reported to be infected with phytoplasma worldwide (Madhupriya 2016).

During a recent survey in five states (New Delhi, Andhra Pradesh, Haryana, Bengaluru, and Pune) of India, phytoplasma suspected symptoms were observed on 17 plants belonging to ten different ornamental plant species (Table 1). Attempts were made to confirm the phytoplasma etiology with the 17 symptomatic plants in the present study by PCR assays, phylogeny, and RFLP analysis.

**Table 1:** Survey, symptoms, locations, and PCR results for phytoplasma detection on ornamental plants

S. no.	Plants and family	Survey period	Location	Symptoms	PCR Results with 3Far/3Rev primer pair and Genbank Acc. No.	Group and Subgroup
1	*Allamanda cathartica* L. (Apocynaceae)	June 2016	IARI, New Delhi	Leaf yellowing	+ve KX641019 +ve KX641020	16Sr VI-D
2	*Bougainvillea glabra* Choisy (Nyctaginaceae)	April 2016	IARI, New Delhi	Leaf yellowing	–	–
3	*Yucca aloifolia* L. (Asparagaceae)	March 2016	DFR, Pune	Leaf chlorosis	–	–
4	*Crossandra infundibuliformis* L. (Acanthaceae)	June 2016	Bengaluru	Leaf yellowing	–	–
5	*Helichrysum italicum* Roth (Asteraceae)	June 2016	IARI, New Delhi	Yellowing and little leaf	–	–
6	*Hibiscus rosa*-*sinensis* L. (Malvaceae)	March 2016	Pune	Leaf yellowing and phyllody	+ve KX641023	16Sr VI-D
7		July 2016	Haryana	Leaf yellowing	–	–
8		July 2016	Andhra Pradesh	Leaf chlorosis	–	–
9	*Tagetes erecta* L. (Asteraceae)	March 2016	Baramati, Pune	Little leaf and witches’broom	–	–
10		April 2016	Andhra Pradesh	Little leaf	–	–
11		May 2016	IARI, New Delhi	Stunting and phyllody	–	–
12		July 2016	Haryana	Yellowing	–	–
13	*Wrightia tinctoria* Roxb. (Apocynaceae)	July 2016	Andhra Pradesh	Leaf chlorosis	–	–
14	*Saponaria officinalis* L. (Caryophyllaceae)	March 2016	DFR Field, Pune	Witches’-broom	+ve KX641021 +ve KX641022	16Sr VI-D
15	*Xanthostemon chrysanthus* (F.Muell.) Benth. (Myrtaceae)	March 2016	Andhra Pradesh	Leaf chlorosis and witches’broom	–	–

*IARI* Indian Agricultural research Institute, New Delhi, *DFR* Directorate of Floriculture Research, Pune

## Materials and methods

### Survey and symptomatology

Surveys of garden, nurseries, and experimental field/plots at New Delhi, Andhra Pradesh, Haryana, Bengaluru, and Pune was made during March to July 2016, and phytoplasma suspected symptoms were collected from 17 plants of ten different ornamental species, viz. *A. cathartica*, *B. glabra*, *Y. aloifolia*, *C. infundibuliformis*, *H. italicum*, *H. rosa*-*sinensis*, *T. erecta*, *W. tinctoria*, *S. officinalis,* and *Xanthostemon chrysanthus* (Table 1).

### DNA extraction

DNA from three healthy and three symptomatic plant tissues (midrib and leaf veins) of 17 ornamental samples were extracted following a described procedure **(**Ahrens and Seemuller 1992). Amplification of phytoplasma ribosomal DNA (rDNA) was performed with the universal phytoplasma primer pairs P1/P7 (Deng and Hiruki 1991; Schneider et al. 1995). Further nested PCR assays were performed with primer pairs 3Far/3Rev (Manimekalai et al. 2010). The DNA isolated from toria phyllody phytoplasma infected *Catharanthus roseus* leaf tissue (Azadvar and Baranwal 2012) was used as positive control.

PCR reactions were carried out in a thermal cycler (Eppendorf, Germany) and the cycling protocol used for the first round PCR using P1/P7 primer pair with initial denaturation at 94 °C for 5 min, followed by 35 cycles consisting of denaturation at 94 °C for 45 s, annealing at 55 °C for 1 min. and extension at 72 °C for 2 min, with the final extension for 10 min at 72 °C. Total PCR mixture (50 μl) contained 100 ng/μl of total nucleic acid, 20 pmol of 3Far/3Rev primers, 1.0 unit of Taq DNA polymerase (G-biosciences), 0.2 mM of dNTP, 2.0 mM MgCl_2,_ and 1X PCR buffer. Two μl of product of the first round of PCR was used in nested PCR using internal primer pairs 3Far/3Rev (Manimekalai et al. 2010). Reaction mixture and condition of nested PCR used were similar as first round PCR except for annealing at 63 °C for 1 min. The PCR product was subjected to electrophoresis in a 1.0% (w/v) agarose gel, stained with ethidium bromide, and observed under UV transilluminator.

### Sequencing and BLAST analysis

The ~1.3 kb nested PCR products were sequenced directly in both directions using 3Far/3Rev primers. The sequences were assembled using DNA baser V.4 program and were further aligned using CLUSTAL W method of Bio-Edit software. Aligned sequences were deposited in NCBI GenBank and used as query sequence in BLASTn search analysis with related submitted sequences in GenBank.

### Phylogenetic analysis

The 16S rDNA sequence generated from the present study and reference phytoplasma strains sequence retrieved from GenBank were used to construct phylogeny by neighbor-joining method with 1000 replications for each bootstrap value using the MEGA 6.0 software version for ‘*Candidatus* Phytoplasma species’ assignment (Tamura et al. 2013). *Acholeplasma laidlawii* was used as out group to root the phylogenetic tree.

### In silico RFLP analysis

The phytoplasma sequences corresponding to the 3Far/3Rev region was subjected to in silico RFLP analysis using pDRAW32 program developed by AcaClone Software (http://www.acaclone.com) and compared with representative sequences of the mollicutes sp. phytoplasma 16Sr VI-D (Ac. No. X83431) subgroup for assigning 16Sr subgroups to ornamental phytoplasma strains analysed by the same restriction mapping utilizing AcaClone software generated RFLP sequences.

## Results and discussion

### Survey and symptomatology

**Fig. 1:**
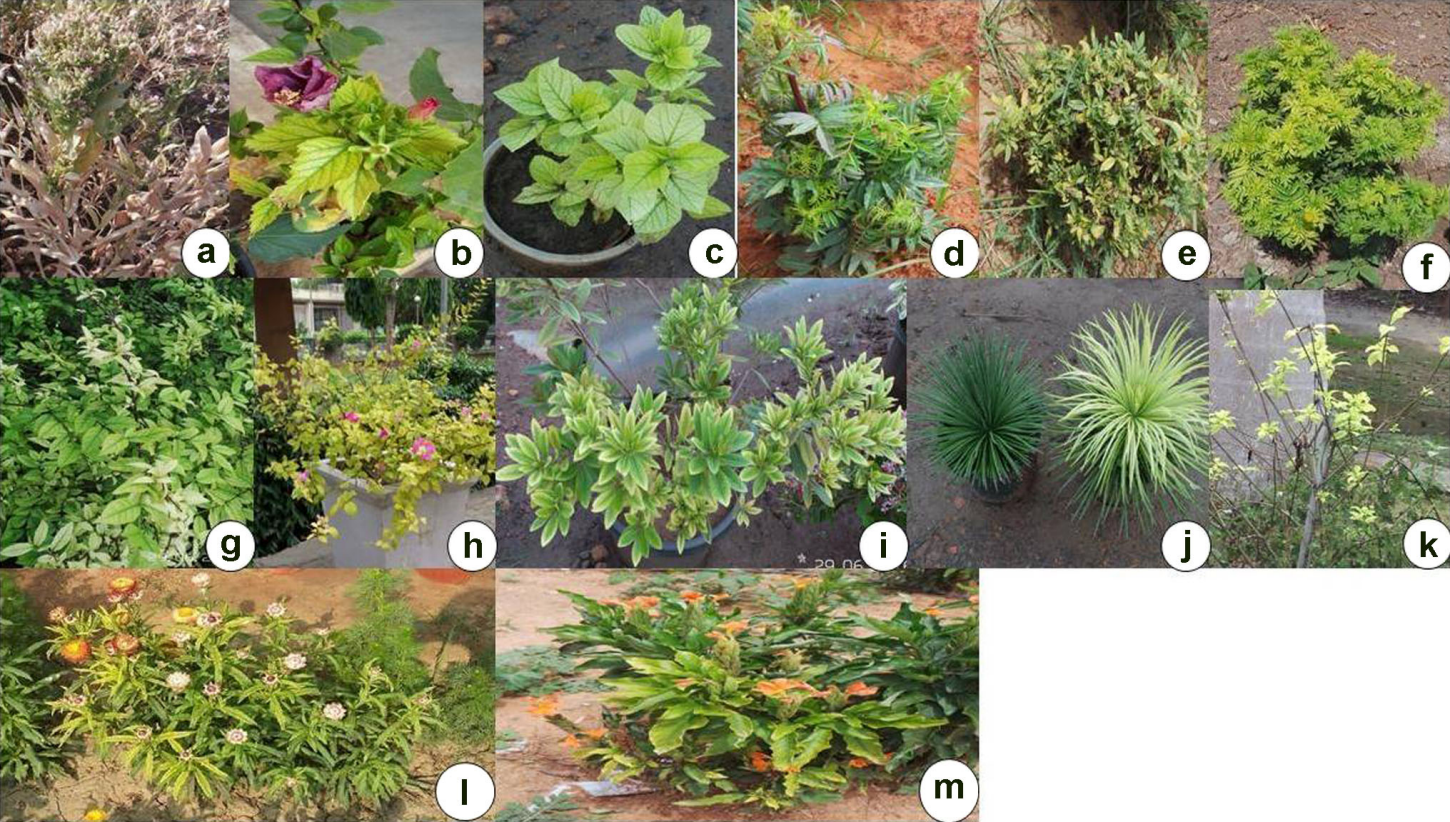
Phytoplasma suspected symptoms on different ornamental plant species **a** Witches’ broom in *Saponaria officinalis* at Pune; **b** Leaf yellowing and phyllody in *Hisbiscus rosa*-*sinensis* at Pune. **c** Leaf chlorosis in *H. rosa*-*sinensis* at Andhra Pradesh. **d** Little leaf in *Tagetes erecta* at Andhra Pradesh; **e** stunting and phyllody in *Tagetes erecta* at Delhi. **f** Little and witches’broom in *Tagetes erecta* at Pune; **g** leaf chlorosis in *Wrightia tinctoria* at Rajahmundary, Andhra Pradesh. **h** Leaf yellowing in *Bougainvillea glabra* at Delhi; **i** leaf chlorosis and witches’broom in *Xanthostemon chrysanthus* at Rajahmundary, Andhra Pradesh, **j** Leaf chlorosis in *Yucca aloifolia* at Pune. **k** Leaf yellowing in *Allamanda cathartica* at Delhi. **l** Leaf yellowing in *Helichrysum italicum* at Delhi. **m** Leaf yellowing in *Crossandra infundibuliformis* at Bengaluru

During survey of garden nurseries and experimental field/plots in New Delhi, Andhra Pradesh, Haryana, Bengaluru, and Pune, phytoplasma suspected symptoms of little leaf, yellowing, chlorosis, phyllody, witches’ broom, and stunting of plants were recorded on ten different ornamental plants species, viz. *A. cathartica*, *B. glabra*, *Y. aloifolia*, *C. infundibuliformis*, *H. italicum*, *H. rosa*-*sinensis*, *T. erecta*, *W. tinctoria*, *S. officinalis,* and *Xanthostemon chrysanthus* (Fig. 1; Table 1).

### Detection of phytoplasma by PCR assays

First round PCR amplification did not yield the expected 1.8 kb product of the 16S rRNA region from any of the 17 symptomatic ornamental test samples with primer pair P1/P7 (data not shown). However, 1.3 Kb amplicons were observed in nested PCR assays with 3Far/3Rev primer pair in five symptomatic plants samples of the three test ornamental species, *H. rosa*-*sinensis* (one isolate, Pune), *S. officinalis* (two isolates, Pune) and *A. cathartica* (two isolates, Haryana) and the positive control of toria phyllody phytoplasma infected *Catharanthus roseus* leaf tissue (Azadvar and Baranwal 2012). No amplifications were observed in any of the rest twelve symptomatic ornamental samples and the non-symptomatic samples (Table 1).

Nested PCR products of five positive amplified products were directly sequenced, and the partial *16Sr RNA* sequences of 1326, 1258, 1333, 1339, and 1252 bp, respectively, were deposited in the GenBank database under the Accession numbers as *A. cathartica* (KX641019, KX641020), *H. rosa*-*sinensis* (KX641023), and *S. officinalis* (KX641021, KX641022) (Table 1).

### Sequence analysis and phylogenetic relationships

**Fig. 2:**
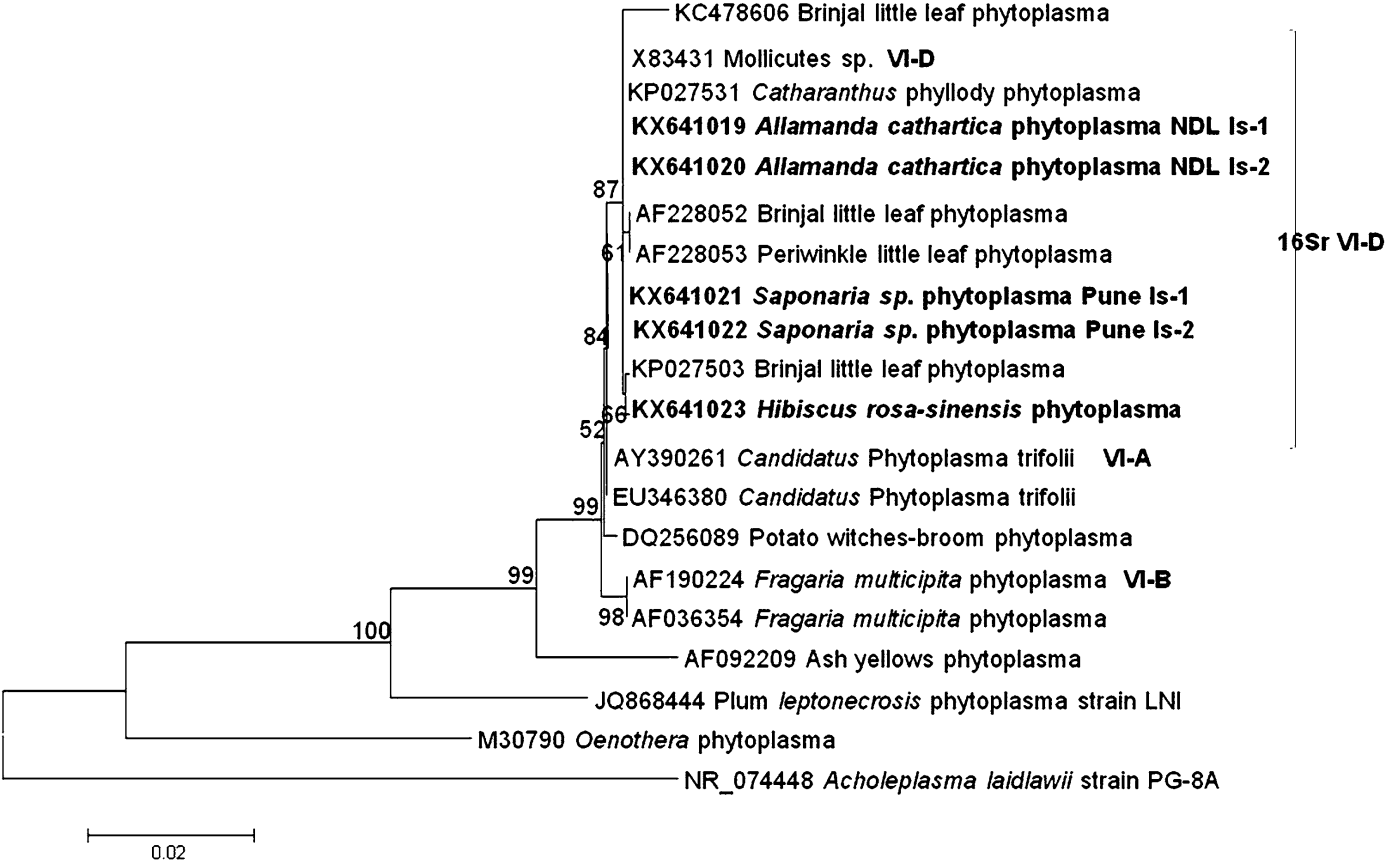
Phylogenetic tree constructed by neighbor-joining method showing the relationships among *H. rosa*-*sinensis*, *S. officinalis* and *A. cathartica*, and the reference phytoplasma strains. Accession numbers are specified in the tree. *A. laidlawii* was used as an out group

Pairwise sequence comparison of the partial *16S rRNA* gene sequences of the five positive ornamental phytoplasma strains of *H. rosa*-*sinensis*, *S. officinalis,* and *A. cathartica* revealed 99% (*H. rosa*-*sinensis* L. Acc No. KX641023) and 100% (*A. cathartica* L. Acc No. KX641019, KX641020; *Saponaria officinalis* L., Acc No. KX641021, KX641022) sequence identity with strains of clover proliferation. Phylogenetic analysis based on 16S rDNA sequences of all the five ornamental phytoplasma strains in the present study revealed their close relationship with strains of clover proliferation group (16SrVI) (Fig. 2).

### In silico RFLP analysis and phytoplasma classification

**Fig. 3:**
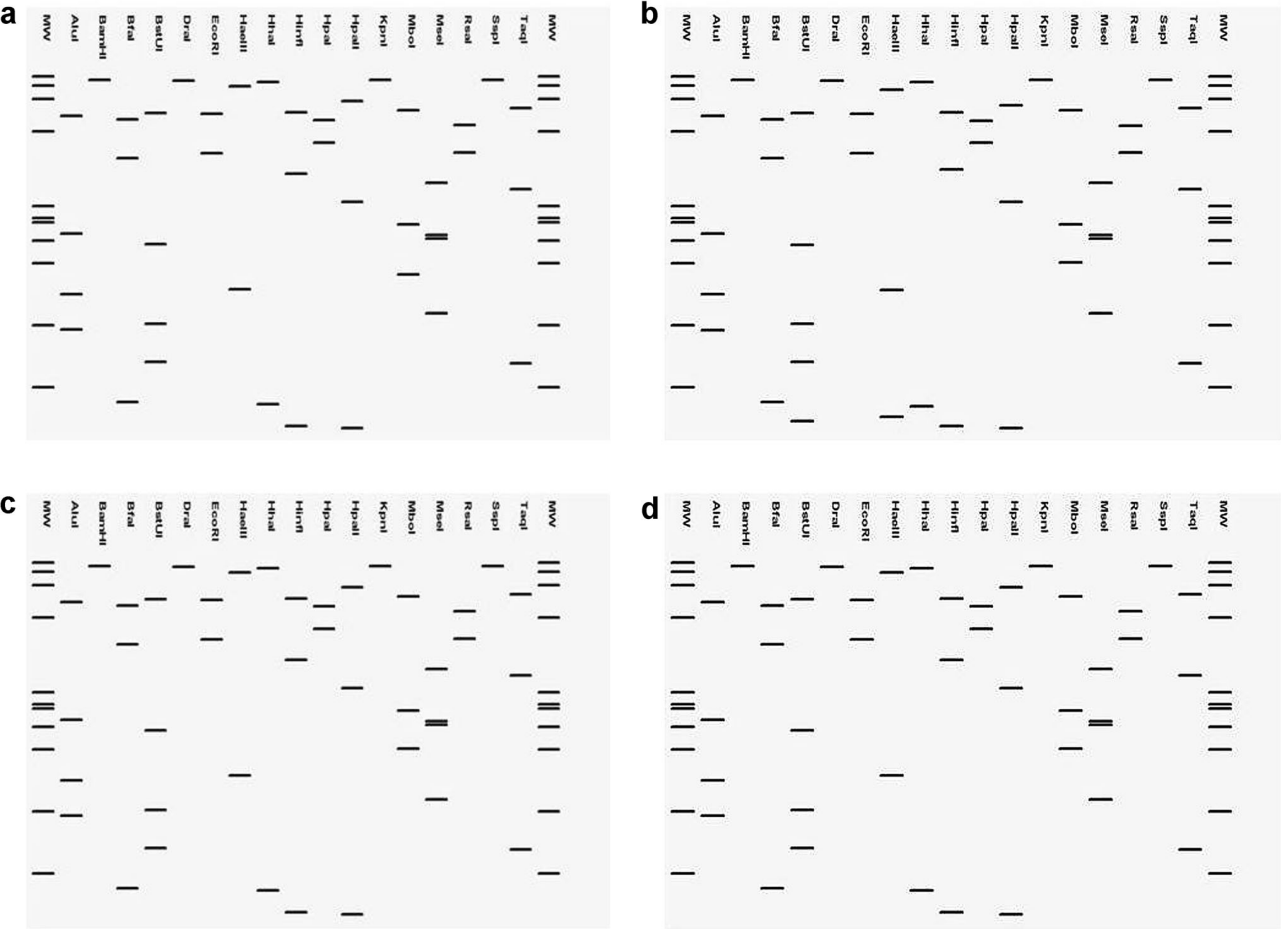
Comparison of virtual RFLP patterns derived from in silico digestions of 1.3 kb 16S rDNA sequences of **a** Brinjal little leaf phytoplasma 16SrVI-D (X83431), **b** *S. officinalis* L. (KX641021), **c** *H. rosa*-*sinensis* L. (KX641023), and **d** *A. cathartica* L. (KX641019) digested using 17 different restriction endonucleases indicating that the *A. cathartica* L., *H. rosa*-*sinensis* L., and *S. officinalis* L. phytoplasma belonged to 16Sr VI-D phytoplasma subgroup

The phytoplasma sequence corresponding to the 3Far/3Rev region was subjected to in silico restriction enzyme digests and virtual gel plotting using the pDRAW32 program developed by AcaClone Software (http://www.acaclone.com). Comparison of restriction site maps with 17 restriction enzymes (*Alu*I, *Bam*HI, *Bfa*I, *Bst*UI (*Tha*I), *Dra*I, *Eco*RI, *Hae*III, *Hha*I, *Hin*fI, *Hpa*I, *Hpa*II, *Kpn*I, *Sau3*AI (*Mbo*I), *Mse*I, *Rsa*I, *Ssp*I, and *Taq*I) through pDraw analysis revealed that the all the three positive ornamental phytoplasma strains (*H. rosa*-*sinensis* KX641023, *A. cathartica* KX641019, and *Saponaria officinalis*, KX641021) produced a virtual RFLP profile identical to phytoplasma reference strain mollicutes sp. phytoplasma (X83431, 16Sr VI-D subgroup) (Fig. 3). Therefore, phytoplasma strains from all the three ornamental plant species in the present study were classified under 16Sr VI-D subgroup.

So far, eleven different groups (16SrI, 16SrII, 16SrIII, 16SrV, 16SrVI, 16SrVII, 16SrIX, 16SrX, 16SrXII, 16SrXIII, and 16SrXIV) of phytoplasmas were identified in ornamental plants in the world (Chaturvedi et al. 2010; Madhupriya 2016). Out of these eleven groups, the 16Sr I group is the most dominant group infecting major ornamental species in India (Chaturvedi et al. 2009; Madhupriya 2016). In the present study, we have reported occurrence of 16SrVI group on three ornamental plants species. In India, 16SrVI group of phytoplasmas was earlier reported to be associated with several diseases of plants, viz. *Araucaria* little leaf (Gupta et al. 2009), brinjal little leaf (Azadvar and Baranwal 2012), *Withania* little leaf (Zaim and Samad 1995; Samad et al. 2006), *Portulaca* little leaf (Samad et al. 2008), *Datura* little leaf (Raj et al. 2009; Singh et al. 2012), *Croton* leaf yellows (Madhupriya et al. 2016), and *Calotropis gigantea* leaf yellows (Madhupriya et al. 2010). However, 16SrVI-D subgroup of phytoplasma was only reported with brinjal little leaf (Azadvar and Baranwal 2012) and *Catharanthus roseus* little leaf (Bertaccini 2015). Hence, in our study, all the three ornamental species are new host records of ‘clover proliferation’ subgroup D in the world. There is a possibility of natural transmission of the 16Sr VI-D subgroup phytoplasma from ornamental species to brinjal crops which is already reported as natural host of 16Sr VI-D subgroup phytoplasma in India (Kumar 2015).
