# Erratum to: Molecular characterization of phytoplasmas of ‘Clover proliferation’ group associated with three ornamental plant species in India

**DOI:** 10.1007/s13205-016-0571-y

**Published:** 2016-11-24

**Authors:** Ekta Khasa, Gopala, Aido Taloh, K. Prabha, Madhupriya, G. P. Rao

**Affiliations:** 1 Division of Plant Pathology, Indian Agricultural Research Institute, Pusa Campus, New Delhi, 110012 India; 2 Directorate of Floricultural Research College of Agriculture, MPKV, Shivajinagar, Pune, 411005 India

## Erratum to: 3 Biotech (2016) 6:237 DOI 10.1007/s13205-016-0558-8

In the original article, one of the co-author’s names has been published incorrectly. The correct name should be K. Prabha.

